# ﻿A new species of freshwater snail of *Fenouilia* (Gastropoda, Pomatiopsidae) from northern Guangxi, China, based on morphological and DNA evidence

**DOI:** 10.3897/zookeys.1196.113856

**Published:** 2024-03-28

**Authors:** Hui Chen, Yue Ming He, Chong Rui Wang, Da Pan

**Affiliations:** 1 Jiangsu Key Laboratory for Biodiversity and Biotechnology, College of Life Sciences, Nanjing Normal University, Nanjing, 210023, China Nanjing Normal University Nanjing China; 2 Hunan Fisheries Science Institute,728 Shuanghe Road, Changsha, 410153, China Hunan Fisheries Science Institute Changsha China

**Keywords:** Diversity, southern China, taxonomy, Triculinae

## Abstract

A new species of pomatiopsid freshwater snail, *Fenouiliaundata* Chen & He, **sp. nov.**, is described from Guangxi, China, based on morphological and molecular evidence. The new species can be distinguished from its congeners by the following combination of characters: shell with low, prosocline, rounded axial ribs and fine spiral striae, broader than high; aperture broader than shell height; radula with lateral teeth have only two or three faint, wavy ridges on inner side. A molecular analysis of partial mitochondrial COI and 16S DNA sequences supports the systematic position of the new taxon.

## ﻿Introduction

Pomatiopsidae Stimpson, 1865 is a family of minute snails with shells usually 1–10 mm high. However, a few species can reach up to 20 mm in high. Typically, pomatiopsids inhabit rivers and streams, while some species also occur in brackish water or even in damp places on land (Liu et al. 1993; Lydeard and Cummings 2019). Shells of pomatiopsids vary in shape from spherical to oval, conical, or tower-shaped (Liu et al. 1979a). The Pomatiopsidae is widely distributed in Asia, South America, North America, Africa, and Australia. It is one of the most species-rich freshwater gastropod families, with approximately 36 recognized genera (Gerlach 2003; Ho and Dang 2010; Lydeard and Cummings 2019). There two main hypotheses about the origin of the Pomatiopsidae are that they originated in either Gondwanaland origin (Davis 1979) or Australia (Attwood 2009); neither of these hypotheses has been completely rejected yet by multilocus phylogenetic analyses (Kameda and Kato 2011; Wilke et al. 2013). The genus and species-level classification are poorly understood (Lydeard and Cummings 2019).

The tropical hills and rivers of China have rich biodiversity and are home to many species of freshwater snails. With at least 18 genera, China has the highest species richness of Pomatiopsidae in the world (Fulton 1904; Kang 1983; Davis et al. 1984; Davis et al. 1990; Davis et al. 1992; Zhang et al. 1997; Xiong and Li 2008; Lydeard and Cummings 2019).

In China, Pomatiopsidae are mainly distributed in the southwestern region (Shu et al. 2014), but the biodiversity of these freshwater snails is likely underestimated, especially in remote regions. Guangxi Province is in southwestern China, and 44 species of freshwater gastropods have been recorded from there (Liang 2023). However, only two genera of Pomatiopsidae have been recorded so far; *Oncomelania* (Gredler, 1881) and *Tricula* (Benson, 1843) occur in mountainous streams in the north of the province (Liu et al. 1979b).

In a recent survey in the Longjiang River, Hechi City, Guangxi, China, a new species of freshwater snail belonging to the genus *Fenouilia* Heude, 1889 was discovered. On comparison of its morphological traits with those of other freshwater snails known from this area, we conclude that this species is indeed undescribed. The new species can be distinguished from its congener *Fenouiliakreitneri* (Neumayr, 1880) by having low, rounded axial ribs on its shell, which is unique to this species. Molecular phylogenetic analyses, based on partial sequences of the mitochondrial 16S rRNA (16S) and COI genes, provide additional evidence supporting the novelty of this species. Our study contributes to a better understanding of pomatiopsid diversity in China and encourages the further exploration of freshwater gastropods in the region.

## ﻿Materials and methods

### ﻿Materials and morphological examination

**Figure 1.**
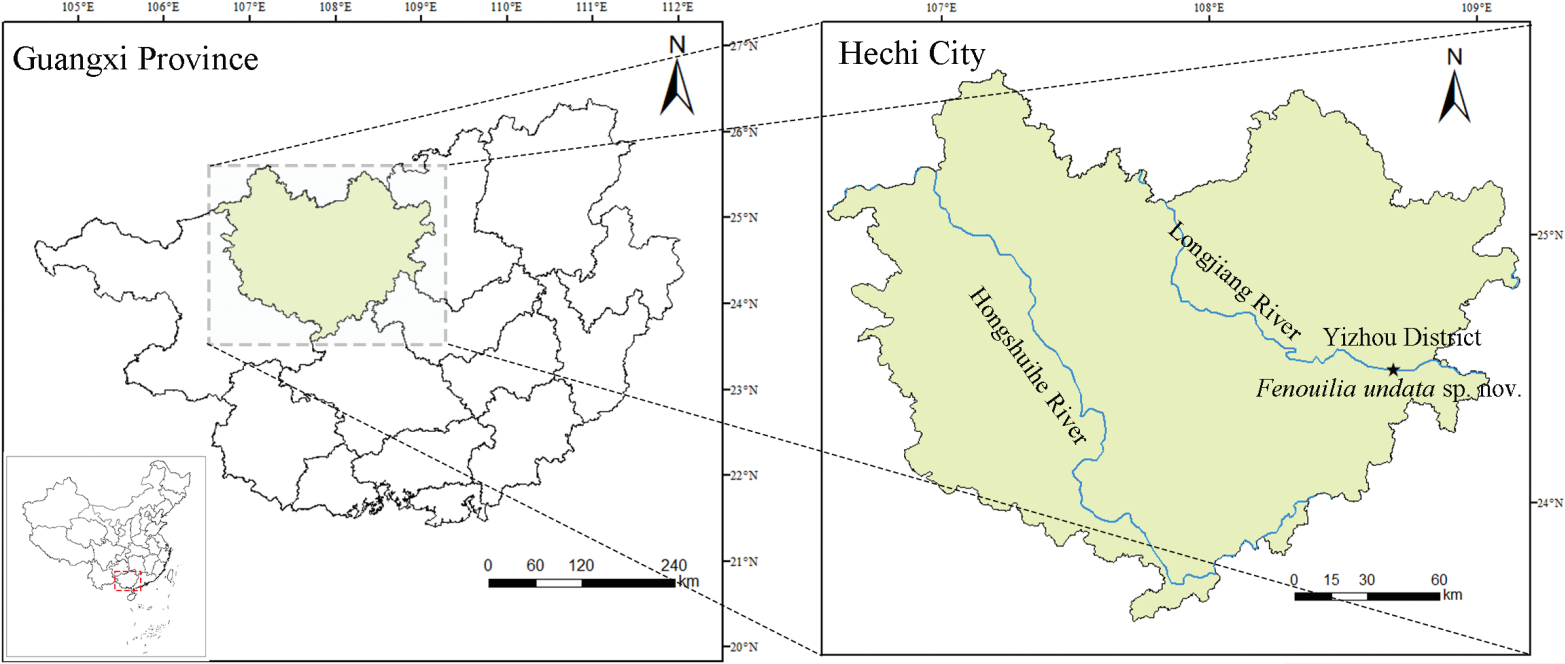
Known distribution of *Fenouiliaundata* sp. nov. (Hechi City), and collection site (Longjiang River).

All specimens were collected by hand in August 2022 and March 2023 on the Longjiang River, Yizhou District, Hechi City, Guangxi Province, China (Fig. 1). They were preserved in 95% ethanol and have been deposited in the Jiangsu Key Laboratory for Biodiversity and Biotechnology, Nanjing Normal University (**NNU**), Nanjing, China.

Before taking any action, preserved samples were soaked overnight in a saline solution. The tissue was extracted using anatomical needles. The shell and operculum were cleaned with a fine brush and then flushed with distilled water and photographed under a Nikon SMZ645 stereomicroscope. For traditional morphometrics, we measured five shell characteristics to the nearest 0.01 mm for each individual using digital calipers as follows: (1) shell height (**H**), which is the maximum dimension parallel to the axis of coiling; (2) shell width (**W**), the maximum dimension perpendicular to H; (3) length of aperture (**LA**), the maximum dimension from the junction of the outer lip with the penultimate whorl to the anterior lip; (4) width of aperture (**WA**), the maximum dimension perpendicular to LA; and (5) height of the body whorl (**BW**), the dimension from the lower margin of the aperture to the upper suture delimiting the first whorl. Other terminology used follows Liu et al. (1979a), Strong (2011), and Du et al. (2019).

Radulae were dissected from the buccal masses of three specimens and cleaned enzymatically with proteinase K following Du and Yang (2023); they were sonicated, mounted on aluminum specimen stubs with adhesive pads, and then observed using a JEOL JSM5610LV scanning electron microscope (**SEM**).

### ﻿DNA extraction, PCR amplification, and phylogenetic analyses

Total genomic DNA was extracted from foot tissue of three ethanol-preserved specimens using a Trelief TM Animal Genomic DNA kit (Tsingke®). Partial sequences of 16S rDNA were amplified using the universal primer set 16Sar CGCCTGTTTATCAAAAACAT and 16Sbr CCGGTCTGAACTCAGATCACGT (Kessing et al. 1989). Partial sequences of COI were amplified using LCO1490 GCTCAACAAATCATAAAGATATT (Folmer et al. 1994) and HCO2198 TAWACTTCTGGGTGKCCAAARAAAT (Glaubrecht and Rintelen von 2003).

Each PCR reaction was performed in a total volume of 20 μL, including 9 μL of PCR mix, 8 μL of double distilled water, 1 μL of each primer and 1 μL of the DNA template. The PCR conditions were as follows: initial denaturation at 95 °C for 3 min; 35 cycles of denaturation at 95 °C for 40 s, annealing at 55 °C for 30 s and extension at 72 °C for 30 s; and final extension at 72 °C for 7 min. Both ends of sequences were obtained by automated sequencing using Applied Biosystems 3730 in Sangon Biotech Co. Ltd (Shanghai, China). In addition, two individuals of *Lithoglyphopsismodesta* (Gredler, 1886) and one of *Fenouiliakreitneri* (Neumayr, 1880) had their 16S rDNA extracted in this study.

To clarify the generic relationship of the new species, we included 16 sequences generated in this study with addition of 16S and COI gene sequences of 49 specimens representing 26 genera and 41 species, which were downloaded from GenBank (Table 1); *Lithoglyphusnaticoides* (L. Pfeiffer, 1828) was used as the outgroup. Sequences obtained in the present study have been deposited in GenBank (for accession numbers, see Table 1). Sequences were aligned using MAFFT v. 7.505 based on the L-INS-i method (Katoh and Toh 2008). Pairwise distances between species were calculated using MEGA X (Kumar et al. 2018). The 16S rDNA and COI were concatenated in PHYLOSUITE v. 2.3 (Zhang et al. 2020).

**Table 1.** Nucleotide compositions of partial 16S rDNA and COI sequences of specimens investigated in this study.

Genus	Species	GenBank		References
		16S	COI	
*Lithoglyphus*	*Lithoglyphusnaticoides*	AF445341	AF445332	Hausdorf et al. 2003
*Bythinella*	*Bythinellaaustriaca*	FJ028832	FJ028942	Benke et al. 2009
	*Bythinellaaustriaca*	FJ028831	FJ028943	
	*Bythinellacarinulata*	FJ028884	FJ029100	
*Paludinella*	*Paludinellaminima*	AB822685	AB822663	Wada et al. 2013
	*Paludinellaminima*	AB822686	AB822664	
	*Paludinellakuzuuensis*	AB822695	AB822675	
*Erhaia*	*Erhaiajianouensis*	AF212894	AF213340	Wilke et al. 2000
	*Erhaiawangchuki*	KY798003	MT237715	Gittenberger et al. 2020
*Akiyoshia*	*Akiyoshiakobayashii*	AB611822	AB611823	Kameda and Kato 2011
*Bithynia*	*Bithyniatentaculata*	FJ160288	JX970605	Wilke et al. 2013
*Pomatiopsis*	*Pomatiopsislapidaria*	AY676118	AF367636	Wilke et al. 2001
*Robertsiella*	*Robertsiella* sp.	AF531548	AF531550	Attwood et al. 2003
*Pachydrobia*	*Pachydrobiamunensis*	KC832721	KC832700	Liu et al. 2014
	*Pachydrobia* sp.	KC832711	KC832690	
*Jullienia*	*Jullieniarolfbrandti*	KC832718	KC832697	
*Hubendickia*	*Hubendickiaschuetti*	KC832709	KC832688	
	*Hubendickiaspiralis*	KC832710	KC832689	
*Jinghongia*	*Jinghongiajinghongensis*	KC832728	KC832707	
*Manningiella*	*Manningiellavelimirovici*	KC832716	KC832695	
	*Manningiellaconica*	KC832719	KC832698	
	*Manningiellapolita*	KC832715	KC832694	
*Tricula*	*Triculabambooensis*	KC832720	KC832699	
	*Triculaludongbini*	KC832717	KC832696	
	*Triculahudiequanensis*	KC832712	KC832691	
	*Triculahongshanensis*	EF394876	EF394896	Guan et al. 2008
*Oncomelania*	*Oncomelaniahupensisrobertsoni*	DQ212900	DQ212855	Wilke et al. 2006
	*Oncomelaniahupensisrobertsoni*	DQ212901	DQ212856	
	*Oncomelaniaminima*	AB611790	AB611795	Kameda and Kato 2011
*Blanfordia*	*Blanfordiaintegra*	AB611722	AB611723	
	*Blanfordiajaponicajaponica*	AB611726	AB611727	
*Cecina*	*Cecinamanchurica*	AB611746	AB611747	
	*Cecinamanchurica*	AB611742	AB611743	
*Neotricula*	*Neotriculaburchi*	AF531542	AF531544	Attwood et al. 2003
	*Neotriculaaperta*	MF663277	MF663265	Attwood et al. 2019
*Gammatricula*	*Gammatriculafujianensis*	AF212896	AF213342	Wilke et al. 2000
	*Gammatriculashini*	AB611798	AB611799	Kameda and Kato 2011
	*Gammatriculachinensis*	EU573993	AF253067	Wilke et al. 2000
*Lacunopsis*	*Lacunopsismunensis*	KC832726	KC832705	Liu et al. 2014
*Delavaya*	*Delavayadianchiensis*	KC832713	KC832692	
*Paraprososthenia*	*Paraprososthenialevayi*	KC832708	KC832687	
*Lithoglyphopsis*	*Lithoglyphopsismodesta*	OR515659	PP327217	This study
	*Lithoglyphopsismodesta*	OR515660	PP327222	
*Fenouilia*	*Fenouiliakreitneri*	OR515658	PP340173	
	*Fenouiliaundata* sp. nov.	OR515661	PP333612	
	*Fenouiliaundata* sp. nov.	OR515662	PP333613	
	*Fenouiliaundata* sp. nov.	OR515663	PP333614	
*Kunmingia*	*Kunmingiakunmingensis*	OR784230	OR780554	
	*Kunmingiakunmingensis*	OR784231	OR780555	

The best-substitution model was selected using the corrected Bayesian information criterion (BIC) in MODELFINDER v. 2.2.0 (Kalyaanamoorthy et al. 2017). For Bayesian analysis, two runs were performed simultaneously with four Markov chains starting from a random tree. Bayesian-inference and maximum-likelihood analyses were performed using MrBayes v. 3.2.7 (Ronquist et al. 2012) and IQTREE v. 2.2 (Minh et al. 2020), respectively, with reference to the selected model of sequence evolution. Bayesian posterior probabilities (BPPs) of nodes were determined using Metropolis-coupled Markov chains (one cold chain) for 2 million generations, with sampling every 1,000 generations. The first 25% of sampled trees were discarded as burn-in when the standard deviation of split frequencies of the two runs was less than 0.01; the remaining trees were then used to create a 50% majority-rule consensus tree and to estimate BPPs. Node support for the maximum-likelihood analysis was determined using 1000 rapid bootstrap (BS) replicates.

Furthermore, to investigate the behavior of the new species, six individuals were maintained in an artificial field environment within the laboratory of Hunan Fisheries Science Institute for one year.

## ﻿Results

**Family Pomatiopsidae Stimpson, 1865**

**Subfamily Pomatiopsinae Stimpson, 1865**

**Genus *Fenouilia* Heude, 1889**

### ﻿ Fenouilia undata

Taxon classificationAnimaliaLittorinimorphaPomatiopsidae

Chen & He sp. nov.

A8AF4EA3-FAE9-55F7-BF5C-EDAC0A3D1037

https://zoobank.org/fffc0cf5-3700-45fe-8905-c9b9698358e0

#### Materials examined.

**Figure 2.**
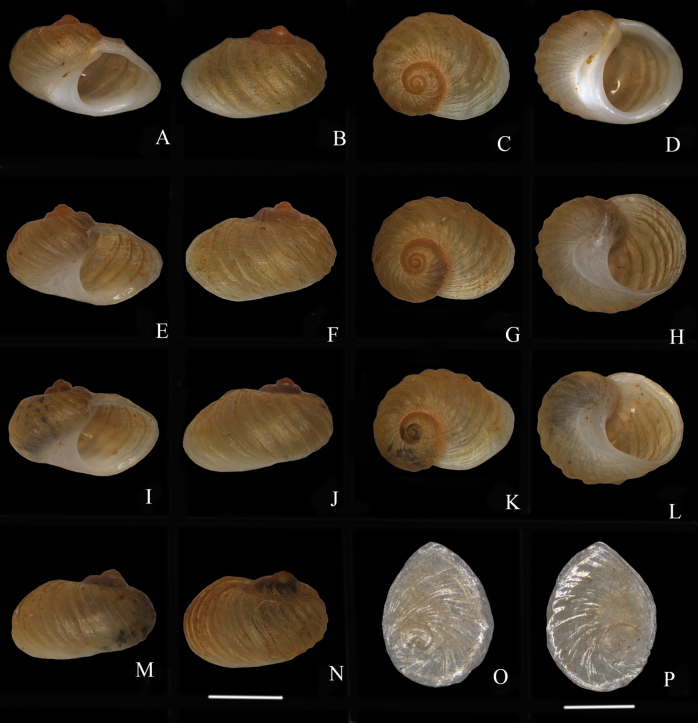
*Fenouiliaundata* sp. nov. shells and operculum **A–D** holotype, NNU230701 **E–H** paratype, NNU230702 **I–L** paratype, NNU230703 **M** paratype, NNU230704 **N** paratype, NNU230705 **O, P** operculum, holotype, NNU230701. Scale bars: 2 mm (**A–N**); 1 mm (**O–P**).

***Holotype***: China • Guangxi Province, Hechi City, Yizhou District, Longjiang River; 24.4927°N, 108.6851°E; August 2022; Xu Cheng Wei & Yue Ming He leg.; NNU230701 (Fig. 2A–D), shell height 3.39 mm. ***Paratypes***: China • 2 specimens; same locality data as holotype; August 2022; NNU230702–03 • _2_ specimens; same locality data as holotype; March 2023; NNU230704–05. Shell height of all paratypes: 3.04–3.44 mm (Fig. 2E–P, Table 2).

**Table 2.** Measurements of *Fenouiliaundata* sp. nov. (in mm). Abbreviations: W, shell width; BW, height of the body whorl; H, shell height; LA, length of aperture; WA, width of aperture.

	Number	H	W	LA	WA	BW
Holotype	NNU230701	3.39	4.95	3.63	2.64	2.84
Paratype 1	NNU230702	3.25	4.67	3.44	2.43	3.07
Paratype 2	NNU230703	3.44	4.68	3.49	2.61	2.92
Paratype 3	NNU230704	3.07	4.37	3.22	2.49	2.81
Paratype 4	NNU230705	3.04	4.01	3.06	2.26	2.74

#### Diagnosis.

Shell small, thin but solid, with rounded, rather flattened shape, and width greater than height; sculptured with low, rounded axial ribs and fine spiral striae; whorls 4–5; body whorl swollen and large. Suture shallow; umbilicus narrow, crescent-shaped or closed. Aperture large, its length greater than shell height. Operculum ovate, corneous, slightly transparent, yellowish.

#### Description.

Shell small, 3.04–3.44 mm high, thin but solid, with a rounded, rather flattened shape; whorls 4–5; body whorl swollen and large, taking up most (about 84–94%) of shell; whorls of spire rapidly expanding. Shell width longer than shell height (Fig. 2). Apex obtuse, usually eroded. Suture low. Shell amber-yellow, with low, prosocline, rounded axial ribs and fine spiral striae. Aperture round, large, broader than shell height. Lip slightly thickened; inner lip smooth, white; outer lip white or yellowish and slightly rolled outward. Umbilicus narrowly crescent-shaped or closed; base white (Fig. 2A–N).

Operculum ovate, smaller than aperture, corneous, thin, slightly transparent, yellowish, length 1.86–2.12 mm, width 1.53–1.72 mm; surface, including nucleus, of operculum smooth; nucleus located at bottom left third (Fig. 2O, P).

**Figure 3.**
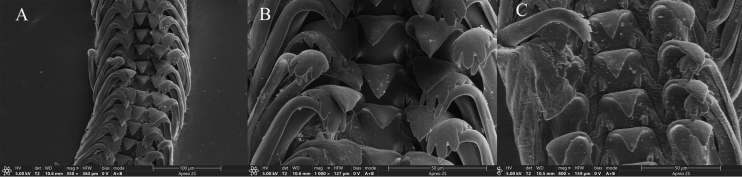
Radula of *Fenouiliaundata* sp. nov. **A** frontal view of radula **B, C** magnified view of radula.

Radula small; ribbon approximately 0.88 mm long. Central tooth with one large, triangular, pointed major cusp without serrations, with two small, sharp cusps on either side at base. Inner side of lateral teeth with two or three faint, wavy ridges; outer side smooth. Inner marginal teeth with five or six small cusps. Outer marginal teeth with 6–8 small cusps (Fig. 3).

**Figure 4.**
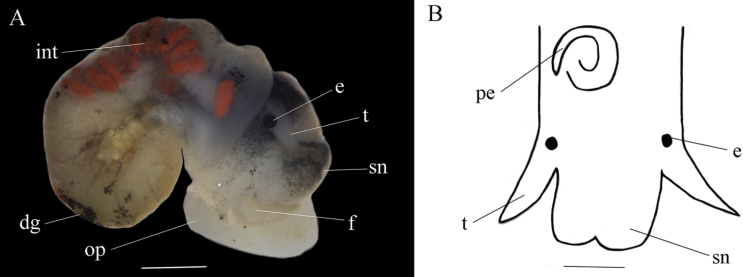
*Fenouiliaundata* sp. nov. **A** dissection with labelled structures of female **B** head of male. Abbreviations: e, eye; t, tentacle; sn, snout; f, foot; op, operculum; dg, digestive gland; int, intestine; pe, penis. Scale bars: 1 mm (**A**); 0,5 mm (**B**).

Tentacles short, white; snout stubby, white, black pigmented. Mantle smooth, light gray, with small black spots. Intestine wider than base of tentacle; digestive gland milky white. Penis translucent white, thin, coiled, located behind right tentacle in neck area (Fig. 4).

#### Habitat and distribution.

**Figure 5.**
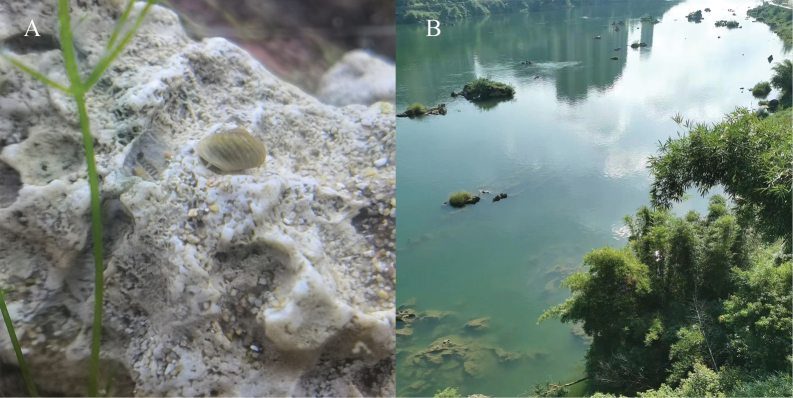
*Fenouiliaundata* sp. nov. **A** color in life **B** natural habitat. Photographs by Xu Cheng Wei and Yue Ming He.

The new species was discovered in the Longjiang River, where the depth of the water was less than 5 m, water flow is variable, and the substrate is composed of large stones (Fig. 5).

#### Biology.

In the laboratory aquarium, the new species fed on algae present on the surface of stones or watergrass. Snails reproduced many times during their year in captivity. Each brownish egg was laid 1.5 mm from the next. Eggs were affixed to the surfaces of rocks with a secretion. In some months, some individuals were observed to occasionally perform a “dance” in which they repeatedly twisted their shells clockwise or counterclockwise. They were more active at night.

#### Remarks.

The genus *Fenouilia* was established by Heude (1889) for *Fenouiliabicingulata* (Heude, 1889) from Dali, Yunnan, China; this species has a trochoidal shell, with rough, raised prosocline growth lines and no umbilicus. Subsequently, Davis et al. (1983) considered *F.bicingulata* to be a synonym of *F.kreitneri* (Neumayr, 1880); thus, the genus was thought to contain only a single species, until now. With prosocline axial ribs, triangular central tooth, and narrowly crescent-shaped or absent umbilicus, the new species is similar to *F.kreitneri*. However, the new species can be distinguished by its broader shell. In addition, *F.undata* sp. nov. has shorter tentacles (vs longer tentacles in *F.kreitneri*), and there are three ridges only on the inner side (vs. lateral teeth with obvious ridges on both sides). The adult shell of *F.undata* sp. nov. is similar to that of *Lacunopsismunensis* (Brandt, 1968) and *Lithoglyphopsismodesta* (Gredler, 1886). These species differ in the relative length of the aperture to shell height (the aperture is longer than shell height in *F.undata*, but shorter in *L.munensis*) and in relative shell width (the shell is broader than height in *F.undata*, but narrower in *L.modesta*).

#### Molecular results.

**Figure 6.**
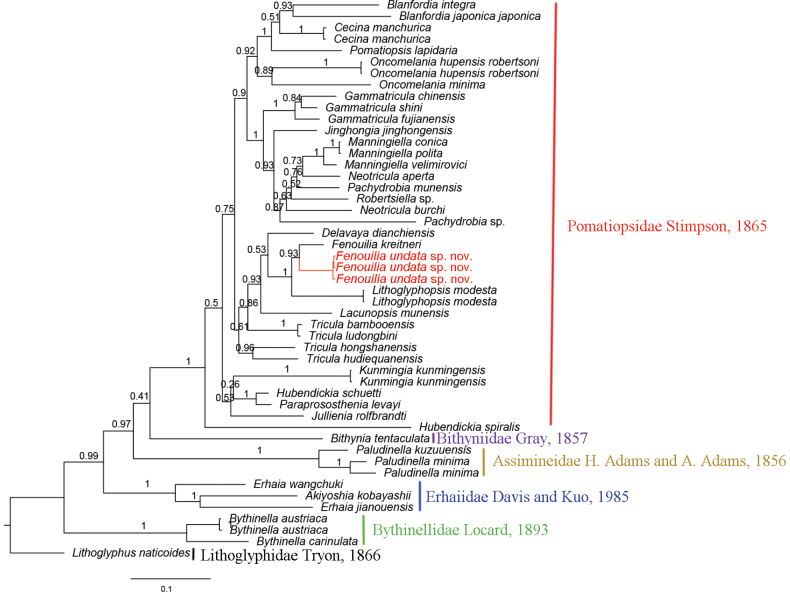
Bayesian-inference (BI) tree inferred from concatenated 16S and COI gene sequences. Posterior probabilities are shown on the left of nodes on the tree.

**Figure 7.**
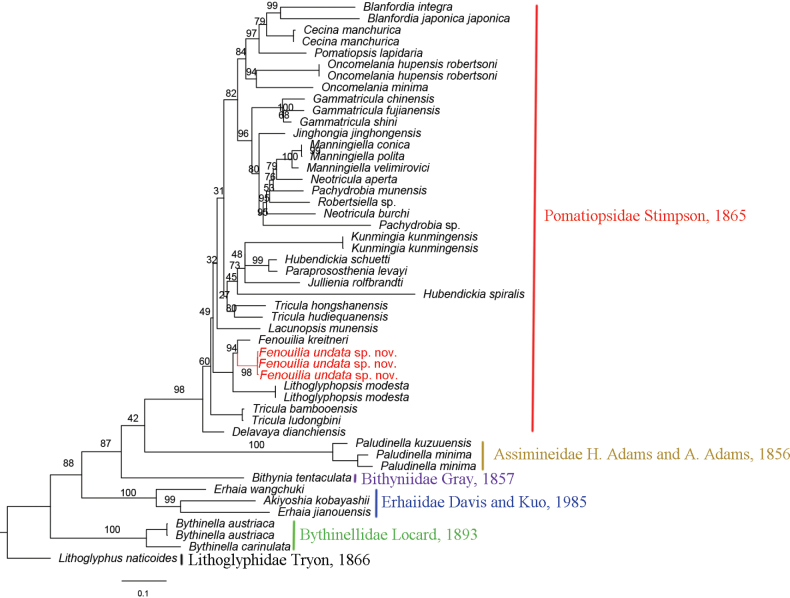
Maximum-likelihood (ML) tree inferred from concatenated 16S and COI gene sequences. Bootstrap supports are shown on the left of nodes on the tree.

The concatenation of COI and 16S rDNA yielded 1229 sites. The GTR+F+R5 model was selected as the best-fit of nucleotide substitution by BIC. Phylogenetic analyses revealed BI and ML trees with largely consistent topologies (Figs 6, 7). The average 16S genetic distance (uncorrected *p*-distance) between the *F.undata* sp. nov. and *F.kreitneri* is 1.04%; the COI *p*-distance is 6.96%.

#### Etymology.

From the Latin adjective *undata* (wavy or wave-like form). We suggest the Chinese common name 波浪龙骨螺.

## ﻿Discussion

The phylogenetic relationships and morphological traits found in this study support the placement of the new species in the genus *Fenouilia*. The prosocline axial ribs on the shell of the new species resemble the prominent, rough, raised prosocline growth lines present in *F.kreitneri*, and the radular of both species has a triangular central tooth without serrated cusps. The molecular phylogenies based on ML and BI analyses show that *Fenouiliaundata* sp. nov. and *F.kreitneri* are nested in a monophyletic group with strong support (BS = 94%, BPP = 0.93) and sister to *Lithoglyphopsismodesta*.

Our phylogenetic tree includes all the genera in China except *Guoia* Davis & Chen, 1992, *Wuconchona* Kang, 1983, and *Parapyrgula* Annandale & Prashad, 1919. The new species can be distinguished from *Wuconchonaniuzhuangensis* (Kang, 1983) and *Parapyrgulacogginiii* (Annandale & Prashad, 1919) by the rounded, flattened shell with its width greater than height. The new species can be distinguished from *Guoiaviridulula* (Möllendorff, 1888) by the presence of axial ribs on the shell and the aperture being longer than the shell height.

The hydrological environment is complex and heterogeneous in southern China. There are still many gaps in surveys for freshwater snails, and more new species have to be discovered. However, the freshwater-snail fauna has been given little attention, especially the small species. With the environmental destruction and habitat modifications, many freshwater snails are gradually disappearing or becoming extinct (Du et al. 2011). Our study highlights the necessity and importance of further field surveys of freshwater snails which will help promote the conservation of freshwater ecosystems. We suggest that further intensified survey efforts are urgently required for accurate understanding of the freshwater snail diversity in southern China.

## Supplementary Material

XML Treatment for Fenouilia undata

## ﻿Additional information

### Author ORCIDs

Hui Chen https://orcid.org/0009-0004-5222-3975

Yue Ming He https://orcid.org/0009-0002-9849-7977

Chong Rui Wang https://orcid.org/0009-0009-2315-6941

Da Pan https://orcid.org/0000-0001-5445-6423

### Data availability

All of the data that support the findings of this study are available in the main text.
